# Estrogen and estrogen receptors in the modulation of gastrointestinal epithelial secretion

**DOI:** 10.18632/oncotarget.18313

**Published:** 2017-05-31

**Authors:** Xin Yang, Yanjun Guo, Jialin He, Fenglian Zhang, Xuemei Sun, Shiming Yang, Hui Dong

**Affiliations:** ^1^ Department of Gastroenterology, Xinqiao Hospital, Third Military Medical University, Chongqing, P.R. China; ^2^ Division of Gastroenterology, Department of Medicine, School of Medicine, University of California, San Diego, California, USA

**Keywords:** estrogen, estrogen receptors, HCO_3_^-^ secretion, Cl^-^ secretion

## Abstract

Gastrointestinal (GI) epithelial ion transport is physiologically important in many aspects of humans, such as in maintaining fluid balance of whole body, and also plays a role in the development and progression of common GI disease. Estrogen and estrogen receptors have been shown to modulate the activity of epithelial ion secretion in GI tract. This review aims to address the current state of knowledge about the role of estrogen and estrogen receptors in modulation of GI epithelial secretion and to elucidate the underlying mechanisms. We highlight the recent findings regarding the importance of estrogen and estrogen receptors in GI epithelia protection and body fluid balance by modulation of gastrointestinal epithelial HCO_3_^-^ and Cl^-^ secretion, especially current information about the regulatory mechanisms of duodenal HCO_3_^-^ secretion based on our study in this field. Since there are no reviews on this topic but only few papers to address the main issues, we hope to timely provide new perspectives for the association between estrogen and GI disease.

## INTRODUCTION

Gastrointestinal (GI) tract secretion plays a critical role in maintaining systemic liquid balance and the progression of several common diseases. Specifically, human GI mucosal HCO_3_^-^ secretion protects GI epithelia against acid-peptic injury. Generally, women suffer less life-threatening and chronic illnesses than men, such as cardio(cerebro)vascular diseases, certain cancers, chronic fibrogenic disorders (atherosclerosis, renal and liver fibrosis), and emphysema [1]. Compared with age-adjusted men, women have lower mortality rates for the 15 leading causes of death [2]. It has been reported over the years that 17b-estradiol (E2) plays a role in maintaining the systemic electrolytes and liquid homeostasis [3–6]. The extra-gonadal effects of estrogen on GI secretions are female-specific and are both genomic and rapid ‘non-genomic’ cell signaling patterns. In GI, the regulation of estrogen in ion channels and transporters appears to have beneficial impact. However, the emergence of the targets and molecular mechanisms related to this modulation is only in the last decade [7, 8].

Although we are on the stage of understanding estrogen role in GI ion transport in general and the effects in health and disease, much rapid estrogen responses in GI is remained to be revealed in the underlying physiological and molecular mechanisms, especially, in the sexual difference in extra-gonadal influence of estrogen on ion channels and transporters. In this review, we highlight the recent findings regarding estrogen in GI secretion, especially intestinal epithelial HCO_3_^-^ secretion based on our study in this field, and hope to provide an insight into the current state of estrogen modulation of GI epithelial secretion and the underlying mechanisms as promising areas for clinical application.

### Estrogen in GI

Estrogens are steroid hormones synthesized in all vertebrates and produced primarily in the reproductive tissues, such as corpus luteum, ovarian follicles and placenta. They are also produced in smaller amount in the non-reproductive tissues, such as breast, liver, adrenal gland, fat tissue and GI as well [9]. There are three types of physiological estrogens: estrone (E1), 17β-estradiol (E2) and estriol (E3). E2 is the most active estrogen, which has profound effects on the growth, differentiation, and function of many reproductive and non-reproductive tissues [9–11]. And it is the primary female reproductive hormone and plays a biological role in target tissues by regulating the transcriptional and cell signaling processes [9]. There is an emerging field of research in the modulation of estrogen in the physiology of non-reproductive tissue. The prominence of absorptive and secretory epithelia among the list of E2 target tissues suggests an important role of estrogen in the regulation of ion transport processes to adjust the body electrolyte and liquid balance [6, 12].

Classically, E2 has been thought to play its role through a genomic mechanism, which mainly combines estrogen with nuclear estrogen receptors and then regulates transcription. The genomic mechanism needs several hours for the effects to be shown as the time required to transcribe and translate estrogen-regulated genes [13]. Nevertheless, rapid responses of E2 found in several cellular systems can’t be classified as a genomic mechanism [14–16]. So these rapid effects were defined as ‘non-genomic’ effects of estrogen [17]. The non-genomic action of estrogen can activate a series of intracellular second messengers rapidly, with potential seconds to minutes in different cells, by interacting with plasma membrane receptors [14–16].

Ueyama et al. reported that E2 levels in the portal vein were much higher than those in arterial blood, and that the main estrogen was determined as E2 with gas chromatography [18]. They also showed the presence of estrogen synthetase, aromatase or cytochrome P450arom in gastric parietal cells. Gastric E2 is synthesized from progesterone via androstenedione and testosterone by enzyme reactions of 17α-hydroxylase (EC: 4.1.2.30), 17β-hydroxysteroid dehydrogenase type 3 (EC: 1.1.1.62) and aromatase (EC: 1.14.14.1), respectively [19]. Thus, a relatively large amount of E2 is synthesized in gastric parietal cells.

As long as gender differences are concerned, sex hormones have been usually considered as the cause factors. For example, many studies have indicated estrogen as a protective role in the development of various diseases, including cardiovascular diseases [20–22], cerebral damage and mortality [23, 24], endometriosis [27, 28] and osteoporosis [25, 26]. Interestingly, in GI, epidemiological studies have also turned out that men have a higher prevalence of peptic ulcer disease than women [29–32], while women taking estrogen-containing oral contraceptive pills or pregnant women exhibit a further reduced frequency of duodenal ulcer [33]. In contrast to ulcer disease, female gender, but not oral contraceptive use, was reported as a risk factor for ulcerative colitis relapse [34]. Overall, it is reasonable to infer that, E2 may have protective effects in the GI tract similar to other organs.

### Estrogen receptors in GI

Estrogens exert their effects via diffusion through the plasma membrane and signaling through hormone-specific estrogen receptors, which are also expressed in the brain, kidney, sweat gland as well as in the respiratory tract [35]. Estrogen receptor (ERα) was first cloned from a human breast cancer cell line in 1986 [36]. Ten years later, a second ER was cloned from rat prostate [37], which was named as ERβ. Most of E2 have their effect through these two nuclear ERs, ERα and ERβ, members of the nuclear receptor super family, which regulate both unique and overlapping physiological effects of E2 [13]. The two nuclear ERs, exhibit distinct transcriptional properties and can form both homodimers and heterodimers [38–40]. In addition, nuclear ERs can sequestrate other DNA-binding proteins as to regulate gene expression in the nucleoplasm.

**Figure 1:**
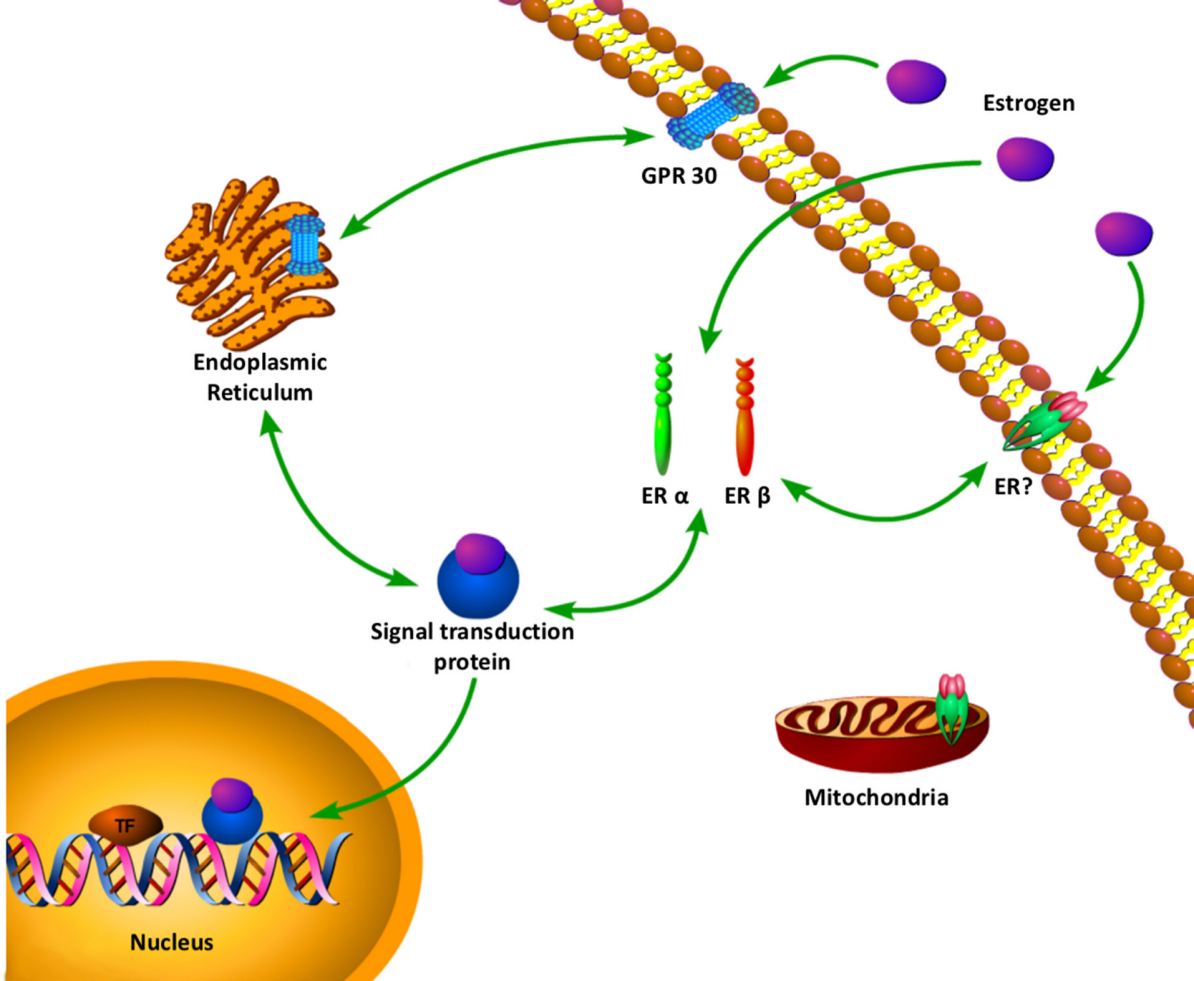
A complex web of estrogen with multiple estrogen receptors signaling has been uncovered Novel location for estrogen receptor and novel rapid crosstalk with other cellular signaling pathways. ERα, Estrogen receptor α; ERβ, Estrogen receptor β; ERE, Estrogen response element; GPR30, G protein-coupled receptor 30.

To add the complexity, a membrane G-protein coupled receptor , GPR30 (or GPER1) has been proposed as an alternative estrogen-binding protein [41, 42]. The first identified alternative estrogen receptor molecularly is GPR30 [41, 43]. In 2000, Filardo et al. first suggested that GPR30 might play a role in the effect of estrogen. They reported that in a breast cancer cell line SKBR3, which only expresses GPR30 but not ERα/β, E2 activates Erk-1/2. A few years later, Thomas et al. showed that E2 was an endogenous ligand for GPR30 located at the plasma membrane [41]. In a quest to better understand the mechanisms, a table and a complex web of estrogen with multiple estrogen receptors signaling has been uncovered, and novel location for estrogen receptor and novel rapid crosstalk with other cellular signaling pathways (Table 1, Figure 1) [44]. It is possible that there are a number of receptors signaling from multiple locations in one cell to give an integrated cellular response to estrogen.

**Table 1:** Basic characteristics of two classes of estrogen receptors

*ESTROGEN RECEPTORS*		
Receptor group	Intracellular receptors	Rhodopsin-like family of seven-transmembrane G protein-coupled receptors
Example	ERα, ERβ	GPR30
Localization	Cytosol, Nucleus	Plasma membrane, Endoplasmic reticulum
Signaling pathway	Genomic	Non-genomic
Ligand	17β-Estradiol (E2)	17β-Estradiol (E2)

ER, estrogen receptor; GPR30, G protein-coupled receptor 30.

The two isoforms of the estrogen receptor, ERα and ERβ, show marked differential tissue expression and biological roles. ERα is predominantly expressed in breast, uterus, vagina and other tissue types [45, 46]. ERβ is predominantly found in ovary, testis, spleen, lung, hypothalamus and thymus with some expression noted in breast tissue [46]. In 2012, a large Chinese cohort study confirmed the existence of ER*α* and ER*β* in both gastric cancer and non-cancer tissues [47]. Recently, Fan Zhou et al. confirmed the presence of ER*α* and ER*β* in stomach by immunohistochemistry [48]. Most of the studies on ERs in GI tract focus on gastric and colonic cancers, but their expression and function are little known in human normal intestinal epithelia. In 2011, our team demonstrated for the first time ER expression in human proximal duodenal mucosae by Western blot analysis and immunohistochemistry. Both ERα and ERβ proteins are expressed at similar levels in the duodenal mucosae of healthy male and female young volunteers and also in mucosal epithelial cells. Of note, in duodenal mucosa, ERs mainly expressed in the cytomembrane and cytosol of the villous and crypt cells [49].

### Modulation of HCO_3_^-^ secretion

A century ago, Russian physiologist Vasilii Boldyreff has described the ability of GI tract to self-regulate luminal acidity [56]. Between the surface epithelium and the acidic lumen can form a pH gradient in chambered gastric or duodenal mucosa *in vitro* [57–59]. The gastric and duodenal mucosal HCO_3_^-^ secretion was one of the main reason caused the pH gradient, which was recognized in the 1970s [60–62]. Gastric and duodenal destruction caused by acid and pepsin are called peptic ulcers (including gastric and duodenal ulcers). Peptic ulcers occur when the balance between aggressive and defensive factors is disrupted. While GI mucosal HCO_3_^-^ secretion makes great effort in GI mucosal protection against acid-induced injury [63, 64]. The proximal duodenal mucosal HCO_3_^-^ secretion is significantly diminished in patients with duodenal ulcer compared to healthy volunteers, which indicates that reduced HCO_3_^-^ secretion plays a role in the pathogenesis of duodenal ulcer [65]. Men suffer duodenal ulcer more often than women. The ratio of male/female in the incidence of duodenal ulcer is 1.9:1 in the United States, 2.2:1 in Europe, and 3.6:1 in China [31, 66, 67]. It suggests that differences between the sexes in duodenal ulcer is likely caused by female sex hormones. Estrogen was shown to prevent peptic ulcer in animals [68, 69], but the mechanisms remain unclear.

Among the pathophysiologic abnormalities in duodenal ulcer vary in frequency, the decreasing of duodenal mucosal HCO_3_^-^ secretion is the most common factor as it protects duodenum against acid-peptic damage. In 2007, we found that basal and acid-stimulated duodenal mucosal HCO_3_^-^ secretion responses were 1.5 and 2.4-fold higher respectively in female than male mice *in vivo* [70]. So our team hypothesized that estrogen stimulates duodenal mucosal HCO_3_^-^ secretion. And we found E2 and also the selective ER agonists of ERα and ERβ, rather than progesterone, stimulated murine duodenal mucosal HCO_3_^-^ secretion. E2 can stimulate murine duodenal mucosal HCO_3_^-^ secretion in both genders, while it has 4.3-fold higher responses in female than in male mice. But neither ERα nor ERβ mRNA and protein expression levels differed according to gender. Our data showed that E2 linked to intracellular calcium, cystic fibrosis transmembrane conductance regulator(CFTR) and Cl^-^/HCO_3_^-^ anion exchanger to stimulate HCO_3_^-^ secretion. It confirmed the differences of the duodenal mucosal HCO_3_^-^ secretion between the sexes in duodenal in mice. And we first found that estrogen stimulated more murine duodenal mucosal HCO_3_^-^ secretion in female than male mice, which may attribute to the lower incidence of duodenal ulcer of clinically observation in premenopausal women than the age-matched men.

In 2011, our experiments conducted an epidemiological investigation on the correlation duodenal ulcer prevalence with sex and age [49]. Proximal duodenal mucosal HCO_3_^-^ secretion was measured from healthy subjects. We found that the prevalence of duodenal ulcer was significantly lower among women than among men. The greatest reduced prevalence occurred between premenopausal women (20–49 years) and age-matched men, which were 3.91–5.09 fold, while among subjects 60 years or older the difference was reduced to ≤ 1.32 fold. Premenopausal (20–29 years), but not post-menopausal (60–69 years) women, had significantly higher basal and acid-stimulated duodenal mucosal HCO_3_^-^ secretion than the age-matched men. However, both ERα and ERβ proteins were expressed at similar levels in the duodenal mucosae of healthy male and female volunteers (20–29 years) by Western blot analysis. It demonstrated that estrogen is beneficial to duodenal ulcer in human beings clinically, but the underlying mechanisms are not explored.

**Figure 2:**
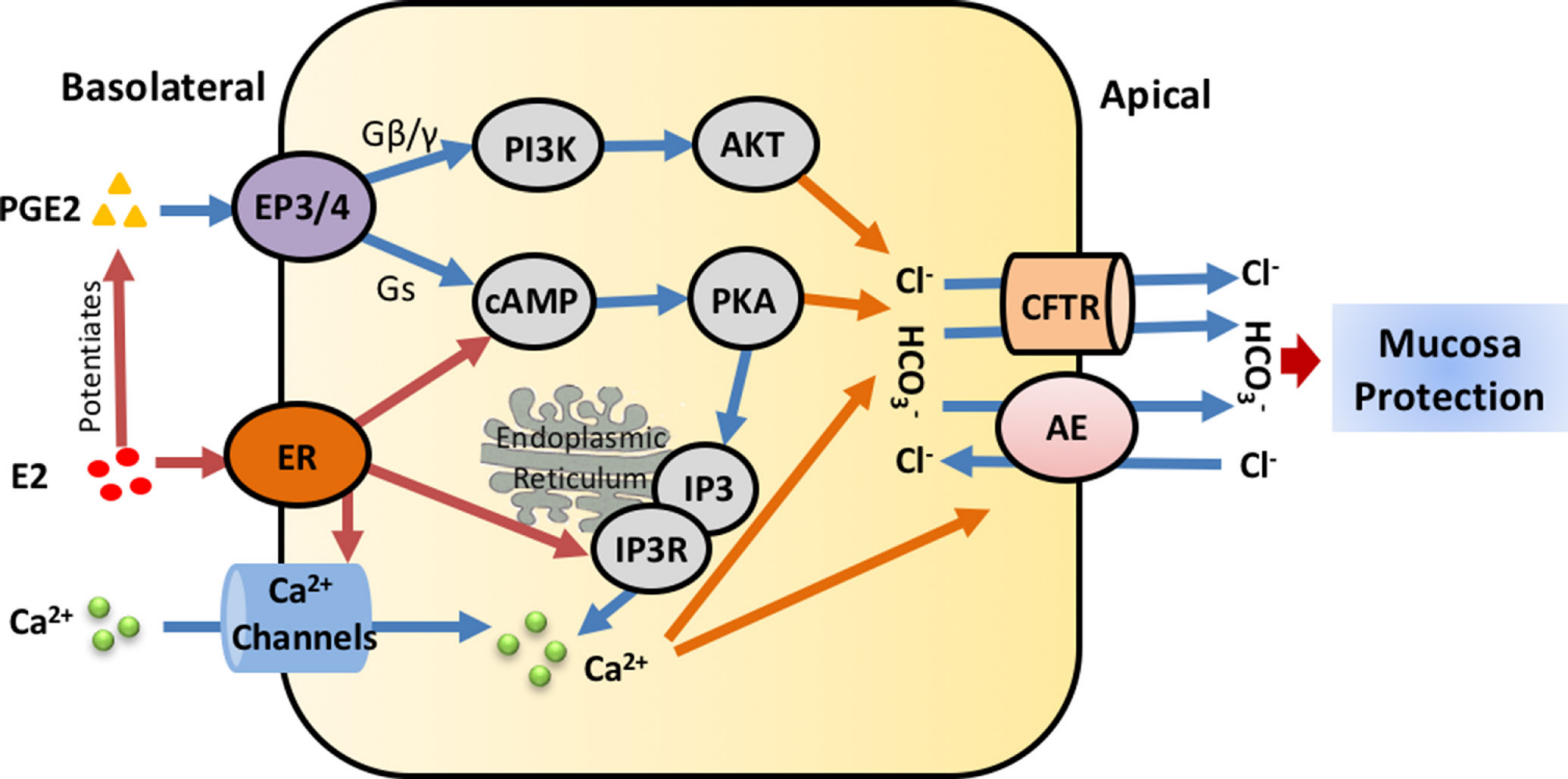
Regulatory mechanisms of estrogen/estrogen receptors on intestinal epithelial ion transports In the duodenal mucosa, estrogen either directly stimulates ER-mediated or potentiates PGE2-mediated epithelial ion transports through Ca^2+^, PKA or AKT signaling pathways, particularly stimulates duodenal mucosal HCO_3_^-^ secretion via CFTR and Cl^-^/HCO_3_^-^ anion exchanger to protect duodenal mucosa. E2, Estrogen; ER, Estrogen receptor; PGE2, Prostaglandin E2; CFTR, cystic fibrosis transmembrane conductance regulator; AE, Cl^-^/HCO_3_^-^ anion exchanger; cAMP, cyclic adenosine monophosphate; PI3K, phosphatidylinositol 3-kinase; IP3R, Inositol 1,4,5-trisphosphate receptor.

Prostaglandins are important mediators of normal physiology. They are members of a family of lipid mediators derived from cyclooxygenase-mediated metabolism of arachidonic acid [71, 72]. Prostaglandin E2 (PGE2), which is distributed widely in the GI tract, can regulate a variety of gastrointestinal functions and is the most important intra-mucosal mediator of acid-stimulated duodenal mucosal HCO_3_^-^ secretion in human beings [73, 74]. PGE2 could increase cAMP production and activate protein kinase A(PKA) that directly stimulates CFTR. And PKA can also phosphorylate IP3 receptors to sensitize them to basal levels of IP3, which could improve IP3 receptor response and enhance Ca^2+^ release from the intracellular stores [75]. In 2012, we found that E2 (at the physiological concentration 1nM) and PGE2 additively increased phosphatidylinositol 3-kinase(PI3K) activity and Akt phosphorylation [76]. And the specific PI3K inhibitor, Wortmannin, can inhibite estrogen-potentiated PGE2-stimulated duodenal mucosal HCO_3_^-^ secretion and *I*sc with Ussing Chamber. In conclusion, Estrogen enhances PGE2-stimulated duodenal mucosal HCO_3_^-^ secretion through the activation of ERα and the cAMP and PI3K-dependent mechanism as Figure 2, which may account to the lower prevalence of duodenal ulcer in young women.

In addition, in 2015, Karim K et al. wanted to elucidate mechanisms underlying differential effects of these hormones on uterine pH. They found high expression of CAII, III, XII, and XIII under the influence of progesterone and estrogen plus progesterone could contribute to the decrease of uterine tissue and fluid pH [77]. However, whether individual estrogen can influence the significance of high levels of CAIX expression remains unclear. And in 2016, Hai Jin1 et al. found endogenous estrogen upregulated the expression and functional activation of CFTR and Cl^-^/HCO_3_^-^ anion exchanger SLC26A6 in duodenal mucosa [70]. All these suggested novel mechanisms of estrogen in regulating HCO_3_^-^ transport.

### Modulation of Cl^-^ secretion

For water conservation, the distal colon is the important site in the human body. The ion transport across the epithelium, especially the Na^+^ and Cl^-^ transport, determines the rate of water loss and recovery from the lumen of the colon. Distal colon has been accounted as a target tissue for E2; indeed both ERα and ERβ were detected in colonic crypts [78].

In 2001, Brian J. Harvey laboratory first described that estrogen inhibits epithelial Cl^-^ secretion of the female distal colon [14]. They found that the activity of K^+^ channel was suppressed after treated with estrogen at physiological levels. The anti-secretory action of estrogen is different between males and females according to the ion transporter protein expression profile. Then in 2011, they found that E2 reduced the currents with Ussing chamber mediated by the KCNQ1:KCNE3 potassium channel rather than KCNQ1:KCNE1 or KCNQ1 alone [79]. And they further revealed that E2 inhibited of intestinal mucosal Cl^-^ secretion induced by enterotoxins [80]. In 2013, Harvey BJ and his colleagues found that E2 can regulate the abundance of KCNQ1 on cell membrane by endocytosis. This study has demonstrated for the first time of hormonal regulation of KCNQ1 trafficking [81, 82]. However, it is unclear if E2 induces this anti-secretory effect via a specific activation of ER, and if so, what subtypes of ERs are involved.

In conclusion, these researches revealed that estrogen physiologically regulated Cl^-^ secretion and contributed to the observed E2-induced water and salt retention during the high estrogen states [83]. As Brian J. Harvey laboratory has published several reviews on their work of this part [77, 84], we just describe briefly here.

### Clinical implications

Estrogens, the primary female sex hormones, were originally characterized through their important role in sexual maturation and reproduction. However, emerging evidence suggests a clinical association between estrogen/estrogen receptors and GI disease, which is certainly an under investigated topic.

Several clinically relevant in vivo models showed that selective stimulation of ERα is sufficient to elicit many biological responses contributed to estrogen action [50–52]. When ERβ agonists were administered to rats with inflammatory bowel disease (IBD), the symptoms of chronic diarrhea were reversed, and intestinal inflammation was reduced [53, 54]. And in 2014, Kumral ZN et al. demonstrated the possible protective effects of ERα and ERβ subtypes in the pathogenesis of colonic and gastric oxidant damage [55]. In the colitis and ulcer groups, both ER agonists and the non-selective E2 reversed the oxidative damage in a similar manner. These findings indicate that estrogen acts through both ERα and ERβ direct and mediated antioxidant mechanisms, where both ER subtypes play equal and efficient roles in the anti-inflammatory action of estrogen, in limiting the migration of neutrophils to the inflamed tissue, reducing the release and activation of cytokines and thereby alleviating tissue damage.

Another clinical implication is the protective effects of estrogen/estrogen receptors in GI disease by hormone replacement therapy (HRT). HRT after menopause was shown to be protective of disease activity in women with IBD, making them less likely to experience a flare of their disease [100]. A study suggests that menopausal hormone therapy users are at a decreased risk of esophageal and gastric adenocarcinoma and also of esophageal squamous cell carcinoma [101]. A large number of preclinical studies show that the expression of the ERβ has an inverse relation of the presence of colorectal polyps and tumors stage, and can mediate a protective response [102].

Genistein is an isoflavone phytestrogen produced naturally in a number of soy products such as soybeans, soy milk, soy flour, and tofu [85], it is structurally related to E2, and has been shown to bind to ER [86]. Recently, genistein is associated with a series of potential health benefits, including the decrease of women bone loss, reduction of clinical symptoms of diabetes and obesity, as well as lower risk of cardiovascular disease, prostate and breast cancer [85, 87–90]. Genistein has also been reported to regulate the functions of many ion transporters, including the Na^+^–K^+^–2Cl^-^ cotransporter [94], CFTR [91, 92], and the K^+^ channels [93]. Although genistein stimulates duodenal mucosal HCO_3_^-^ and Cl^-^ secretion by activating the CFTR channels, the activation of the HCO_3_^-^ and Cl^-^ secretion of CFTR channels in the duodenal epithelium are different. Since we have demonstrated that genistein is a greater activator of CFTR HCO_3_^-^ conductance than Cl^-^ conductance [95], we therefore propose that it could be clinically used for human GI epithelial protection. Additionally, genistein is promising pharmacotherapeutic for cystic fibrosis by modulating of ion transporters involved in the Cl^-^ and HCO_3_^-^ secretion of specific epithelial tissues [91, 92, 96].

Gender differences in gastrointestinal motility are known in state of health and disease, likely due to the effects of female hormones. Estrogen and progesterone receptors are found throughout the GI tract and may affect its motility [97]. Men have increased stomach acid production and more physiologic gastro-esophageal reflux than women [98]. All these sex differences may affect diagnostic parameters and therapeutic strategies of upper GI dysmotility. And it was also reported that estrogen receptor ligands modulate colonic motility and visceral pain [99].

As discussed earlier, since estrogen physiologically potentiates duodenal mucosal HCO_3_^-^ secretion, it may play an important role in the protection of GI mucosa against acid-induced injury. In distal colonic crypts, estrogen inhibits epithelial Cl^-^ secretion in the female colon, which may prevent diarrhea. It was generally thought the regulatory mechanisms of HCO_3_^-^ and Cl^-^ secretion in GI tract are very similar. However, our study with others revealed that estrogen regulation of GI epithelial HCO_3_^-^ and Cl^-^ secretion is obviously different, which stimulates epithelial HCO_3_^-^ secretion but inhibits Cl^-^ secretion. Although the detailed underlying mechanisms need further investigation, the unique roles of estrogen in GI protection and diarrhea prevention may indicate a new perspective for pharmacotherapeutic development.

## CONCLUSIONS

Previously, the interaction between organs have been reported between the reproductive system and urinary, intestinal systems [103]. However, the GI has not been considered as a sex-steroid targeted organ. The identification of functional ER in GI indicates the physiological roles of estrogen and ER in this system [78]. We demonstrated for the first time that estrogen regulation of duodenal mucosal HCO_3_^-^ secretion may contribute to the protection of duodenal mucosa from acid-induced damage and reducing the risk of duodenal ulcer formation. However, in the colon, E2 can decrease Cl^-^ secretion. The unique roles of estrogen in GI protection and diarrhea prevention may be beneficial for potential pharmacotherapeutic development. HRT by using genistein might have a favorable effect on GI mucosal protection. Further study of estrogen/ERs in the digestive system could yield novel targets for the prevention and treatment of GI disease.

## Abbreviations

GI: gastrointestinal
PI3K: phosphatidylinositol 3-kinase
CFTR: the cystic fibrosis transmembrane conductance regulator
CA: carbonic anhydrase
IBD: inflammatory bowel disease
PKA: protein kinase A
HRT: hormone replacement therapy
PGE2: Prostaglandin E2
